# Editorial: CO_2_ capture and conversion: advanced materials and processes

**DOI:** 10.3389/fchem.2023.1310024

**Published:** 2023-10-24

**Authors:** Zhangfeng Shen, Huayang Zhang, Yangang Wang

**Affiliations:** ^1^ College of Biological Chemical Science and Engineering, Jiaxing University, Jiaxing, Zhejiang, China; ^2^ School of Chemical Engineering and Advanced Materials, The University of Adelaide, Adelaide, Australia

**Keywords:** CO_2_ capture, CO_2_ conversion, catalytic materials, theoretical calculation, advanced technologies

The continuously increase of the atmospheric carbon dioxide (CO_2_) concentration have triggered global warming and climate change, and carbon neutrality is one of the most important target for human society. CO_2_ capture and conversion have become hot areas of research and development aimed at mitigating climate change and reducing greenhouse gas emissions. Advanced materials and processes play a crucial role in these efforts.

In CO_2_ capture, the goal is to capture CO_2_ emissions from various sources such as power plants, industrial processes, and transportation. Advanced materials like porous materials, membranes, and solvents are being developed to selectively capture CO_2_. These materials have high surface areas and specific properties that enable efficient CO_2_ adsorption and separation. Karolina form West Pomeranian University of Technology prepared carbonaceous materials from beet molasses through a hydrothermal process followed by chemical activation and used them for CO_2_ capture (Kielbasa). The activated biocarbon with a high specific surface area of 2005 m^2^g^−1^ and a total pore volume of 0.851 cm^3^g^−1^ gave the highest CO_2_ adsorption of 7.1 mmol/g at 1 bar and 0 °C.

Once CO_2_ is captured, it can be converted into valuable products through various processes. Advanced catalytic materials are being explored to convert CO_2_ into chemicals, fuels, and other useful products. For example, CO_2_ can be converted into methanol, which can be used as a fuel or as a feedstock for the production of other chemicals. Xu et al. from Jiangsu University prepared a Cu_1_In_2_Zr_4_-O-C catalyst with Cu_2_In alloy structure by sol–gel method and used for CO_2_ hydrogenation to methanol (Song et al.). They found the plasma treatment before or after calcination could improve the CO_2_ hydrogenation activity to some extent. Especially, a CO_2_ conversion of 13.3%, a methanol selectivity of 74.3%, and a CH_3_OH space-time yield of 3.26 mmol/gcat/h could be achieved on the catalyst of Cu_1_In_2_Zr_4_-O-PC, which was plasma-modified before calcination, under the conditions of reaction temperature 270 °C, reaction pressure 2 MPa, CO_2_/H_2_ = 1/3, and GHSV = 12,000 mL/(gh). This because the plasma modification can reduce the particle size, enhance the interaction between Cu and In, and shift the Cu 2p orbital binding energy to a lower position. It is expected that advanced technologies will make great contribution in the preparation of materials, which have high CO_2_ conversion efficiency and stability.

Electrochemical processes, such as electroreduction, are also being investigated for CO_2_ conversion. Cao et al. from Jiaxing University reviewed the recent progress of electrocatalytic CO_2_ reduction reaction (CO_2_RR) related to different types of single-atom catalysts (SACs) and double-atom catalysts (DACs) from the perspective of theoretical calculation (Meng et al.). They summarized different catalytic reaction mechanisms of SACs and DACs, and studied the influences of solvation and electrode potential on CO_2_RR to simulate the real electrochemical environment. In addition, they designed three types of Cu-based catalysts (Cu@CNTs, Cu_4_@CNTs, and CuNi_3_@CNTs) and compared their structure towards the activity of electrocatalytic CO_2_RR (An et al.). It was found that the CuNi_3_@CNTs show the largest CO_2_ adsorption energy (−0.82 eV), improving the CO_2_RR selectivity compared with hydrogen evolution, and the CO_2_RR product changes from CH_4_ to CO with a size increase from single-atom Cu to the Cu_4_ cluster. The Cu4@CNTs displayed an extremely low overpotential of 0.02 V for CO formation, while Cu@CNTs showed the highest selectivity for CH_4_ formation among the three catalysts. Theory and computation will play an important role in discovering new materials for CO_2_ conversion since the data-driven methods have been widely applied in material design.

In addition to materials, advanced processes are being developed to enhance the efficiency and scalability of CO_2_ capture and conversion technologies, although we did not receive relevant article in this Research Topic. These include process intensification, integration of renewable energy sources, and optimization of reaction conditions. It is noteworthy that while CO_2_ capture and conversion technologies have the potential to contribute to greenhouse gas reduction, they should be considered as part of a comprehensive strategy that includes energy efficiency, renewable energy deployment, and sustainable practices.

Despite the progress achieved to date, there is great need for further research to be conducted. For example, there is limited understanding of mechanism of interaction between CO_2_ and catalyst surface and more fundamental research is needed to achieve optimal catalyst designs, particularly those materials coupling with the kinetic and thermodynamic requirements of CO_2_ reduction. Overall, the development of advanced materials and processes for CO_2_ capture and conversion is an important step towards achieving a low-carbon and sustainable future.

